# Supplementary material for: New Insights into Lichenization in Agaricomycetes Based on an Unusual New Basidiolichen Species of Omphalina s. str
Source: J Fungi (Basel). 2022 Sep 29;8(10):1033. doi: 10.3390/jof8101033 (PMC9605393; doi:10.3390/jof8101033)
Supplement: Supplementary file 1 [file jof-08-01033-s001.zip › Supplementary Materials.pdf]

**Table S1.** Specimens used for DNA extraction and GenBank accession number of all samples used in this study

| Species | Voucher information | GenBank Accession numbers | |
|---|---|---|---|
| | | ITS | nu LSU |
| *Acantholichen campestris* | DIC595b | KT429798 | KT429810 |
| *Acantholichen variabilis* | MDF679 | KT429796 | KT429808 |
| *Arrhenia discorosea* | G1072 | — | MK278416 |
| *Arrhenia epichysium* 1 | WU_21938 | MW113695 | — |
| *Arrhenia epichysium* 2 | Redhead 5223 | U66442 | — |
| *Arrhenia epichysium* 3 | G1952 | — | MK277599 |
| *Arrhenia fusconigra* 1 | GB-0065942 | MH198228 | — |
| *Arrhenia fusconigra* 2 | GB-0065958 | MH198229 | — |
| *Arrhenia peltigerina* | Diederich 16735 (LUX) | — | MW882126 |
| *Arrhenia rustica* | G1220 | — | MK278422 |
| *Arrhenia sphagnicola* | G1957 | — | MK277602 |
| *Chrysomphalina aurantiaca* 1 | UBC:F34015 | ON738529 | — |
| *Chrysomphalina aurantiaca* 2 | iNAT:9391403 | ON212944 | — |
| *Chrysomphalina aurantiaca* 3 | UBC:F33453 | MN954723 | — |
| *Chrysomphalina chrysophylla* 1 | K(M):233320 | MZ159640 | — |
| *Chrysomphalina chrysophylla* 2 | iNAT:9986321 | MH256117 | — |
| *Chrysomphalina grossular* 1 | OSC 113683 | EU644704 | EU652373 |
| *Chrysomphalina grossular* 2 | OSC 113667 | — | EU652372 |
| *Chrysomphalina grossular* 3 | iNat100045557 | ON856371 | — |
| *Contumyces rosellus* | MGW1462 | — | MF318912 |
| *Cora byssoidea* | DIC151 | KF443234 | KF443258 |
| *Cora inversa* | DIC149 | KF443236 | KF443260 |
| *Cyphellostereum imperfectum* | DIC115a | KF443218 | KF443243 |
| *Cyphellostereum phyllogenum* | DIC158 | KF443219 | KF443244 |
| *Dictyonema hernandezii* | DIC122 | KF443221 | KF443246 |
| *Dictyonema obscuratum* | DIC126 | KF443223 | KF443249 |
| *Gerronema indigoticum* 1 | HMJAU47636 | MK693727 | — |
| *Gerronema indigoticum* 2 | HMJAU47942 | MK693728 | — |
| *Gerronema indigoticum* 3 | HMJAU47636 | NR166278 | NG067873 |
| *Gerronema keralense* | CAL1666 | NR159832 | NG064531 |
| *Gerronema kuruvense* 1 | CAL1665 | — | NG064530 |
| *Gerronema kuruvense* 2 | BKF10266 | MZ452090 | MZ452669 |
| *Gerronema nemorale* 1 | FA236 | MN744687 | — |
| *Gerronema nemorale* 2 | FA239 | MN744688 | — |
| *Haasiella splendidissima* 1 | JVG1071013-1 | JN944395 | JN944396 |
| *Haasiella splendidissima* 2 | Herbarium Roux n. 3666 | JN944398 | JN944399 |
| *Haasiella splendidissima* 3 | Herbarium Roux n. 4044 | JN944400 | JN944401 |
| *Haasiella venustissima* 1 | A. Gminder 971488 (STU) | KF291092 | KF291093 |
| *Haasiella venustissima* 2 | E.C. 08191 | JN944393 | JN944394 |
| *Infundibulicybe alkaliviolascens* 1 | HMJU509 | MW880700 | MW880706 |
| *Infundibulicybe alkaliviolascens* 2 | KUN-HKAS 115933 (Zhao2801) | MZ855883 | MZ853569 |

| | | | |
|---|---|---|---|
| *Infundibulicybe catinus* | — | HM631720 | — |
| *Infundibulicybe costata* | G0484 | — | MK278217 |
| *Infundibulicybe geotropa* | ALV4344 | KT122792 | KT122793 |
| *Infundibulicybe gibba* 1 | KUN-HKAS 92032 (Cai 1412) | MZ675563 | MZ675574 |
| *Infundibulicybe gibba* 2 | KUN-HKAS 73336 (Qin350) | MZ718998 | MZ719010 |
| *Infundibulicybe hongyinpan* 1 | KUN-HKAS 105573 (JSP 248) | MZ718999 | MZ719011 |
| *Infundibulicybe hongyinpan* 2 | HBAU15234 | MW862268 | — |
| *Infundibulicybe kotanensis* | LAH35902 | MN017278 | — |
| *Infundibulicybe mediterranea* | — | HM631724 | — |
| *Infundibulicybe rufa* 1 | KUN-HKAS 57811 (Yang 5268) | MZ719001 | MZ719013 |
| *Infundibulicybe rufa* 2 | KUN-HKAS 77865 (Qin 464) | MZ719000 | MZ719012 |
| *Lichenomphalia altoandina* 1 | SGO:160478 | KT371534 | KT371535 |
| *Lichenomphalia altoandina* 2 | SGO:160478 | NR158477 | — |
| *Lichenomphalia alpina* | O-L-195732 | KY266895 | — |
| *Lichenomphalia grisella* | KH73 | GU234100 | — |
| *Lichenomphalia hudsoniana* 1 | GAL18249 | JQ065873 | JQ065875 |
| *Lichenomphalia hudsoniana* 2 | Gulden247/86 | JQ065874 | — |
| *Lichenomphalia hudsoniana* 3 | K(M):168207 | MZ159424 | — |
| *Lichenomphalia hudsoniana* 4 | 11-32112 (KUN-L) | KY435909 | — |
| *Lichenomphalia lobata* 1 | Palice 2327 | AY542866 | — |
| *Lichenomphalia lobata* 2 | Palice 3275 | AY542867 | — |
| *Lichenomphalia luteovitellina* (*Omphalina luteovitellina*) | — | AY293962 | — |
| *Lichenomphalia meridionalis* 1 | S-270-FB1 | LC428308 | LC428307 |
| *Lichenomphalia meridionalis* 2 | A1368 | MT035854 | MT032349 |
| *Lichenomphalia umbellifera* 1 | GAL15152 | GU810926 | GU811011 |
| *Lichenomphalia umbellifera* 2 | — | AY293959 | — |
| *Lichenomphalia umbellifera* 3 | — | AY293961 | — |
| *Lichenomphalia umbellifera* 4 | 12-36882 (KUN-L) | KY435923 | — |
| *Lichenomphalia umbellifera* 5 | 12-34741 (KUN-L) | KY435917 | — |
| *Loreleia marchantiae* 1 | Y. Makinen 87-190 | MF319070 | — |
| *Loreleia marchantiae* 2 | M. Lahti 24/14 | — | MF318926 |
| *Omphalina chionophile* 1 | GG106_88 | GU234144 | — |
| *Omphalina chionophile* 2 | CBS 553.91 | MH862276 | MH873957 |
| *Omphalina rivulicola* 1 | CBS 558.87 | MH862099 | MH873788 |
| *Omphalina rivulicola* 2 | CBS 560.87 | MH862101 | — |
| *Omphalina pyxidate* 1 | R. Saarenoksa 48384 | MF319071 | MF318927 |
| *Omphalina pyxidate* 2 | TO AV98 | JN944402 | JN944403 |
| *Omphalina pyxidate* 3 | GT99398 | JQ671000 | — |
| *Omphalina pyxidate* 4 | EM0434-05 | JQ671001 | — |
| ***Omphalina licheniformis* sp. nov. 1** | **JX001 (HMAS-L 154705)** | **ON723778** | **ON723776** |
| ***Omphalina licheniformis* sp. nov. 2** | **ZRL20220005 (HMAS 281952)** | **ON723779** | **ON723777** |
| *Rickenella mellea* 1 | — | U66438 | — |
| *Rickenella mellea* 2 | CBS 581.87 | MH862107 | MH873796 |
| *Trogia benghalensis* | CUH:AM031 | KU647630 | — |

| | | | |
|---|---|---|---|
| *Trogia infundibuliformis* 1 | N.K.Zeng2233 | MT822925 | MT829108 |
| *Trogia infundibuliformis 2* | KUN_HKAS56709 | JQ031776 | JQ031781 |
| *Trogia venenata* 1 | KUN_HKAS56679 | JQ031773 | JQ031779 |
| *Trogia venenata 2* | KUN_HKAS54710 | JQ031772 | JQ031778 |
| *Multiclavula petricola*（outgroup） | H. Masumoto 356 ex-type | LC516464 | LC516465 |
| *Multiclavula caput-serpentis*（outgroup） | KaiR699 | MW386064 | MW369074 |
| *Multiclavula corynoides*（outgroup） | Lutzoni 930804-2, DUKE | U66440 | — |
| *Multiclavula vernalis*（outgroup） | Lutzoni 930806-1, DUKE | U66439 | — |

Notes: Newly generated sequences are in bold font. '—' indicates that the corresponding information or sequence is absent.

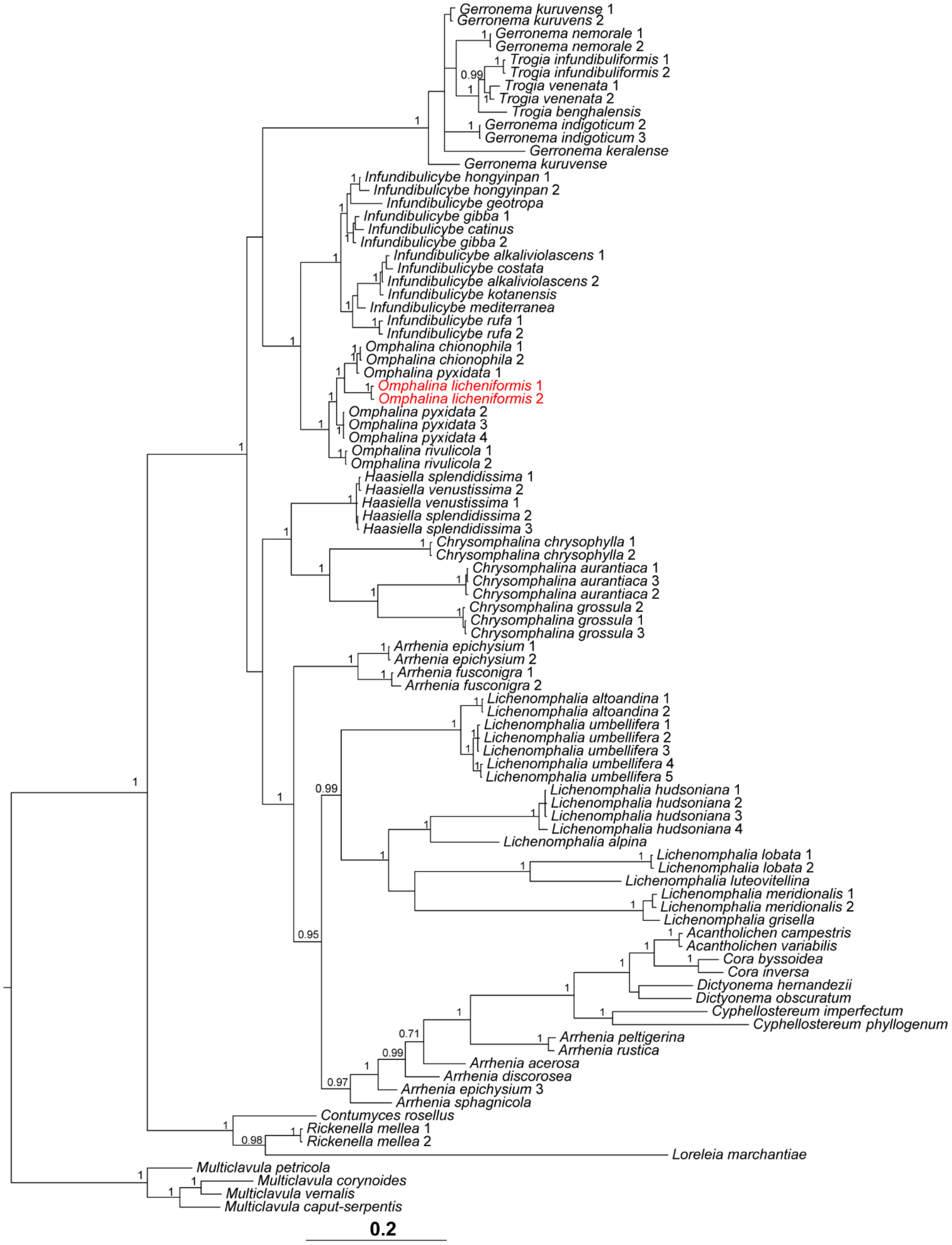


**Figure S1.** The Bayesian tree based on the concatenated ITS + nuLSU data sets. The numbers in each node represent posterior probability (PP) values. PP values ≥ 0.95 were plotted on the branches of the tree. The samples in red color indicate that these sequences were newly generated for this study. Scale in 0.2 substitution per site.

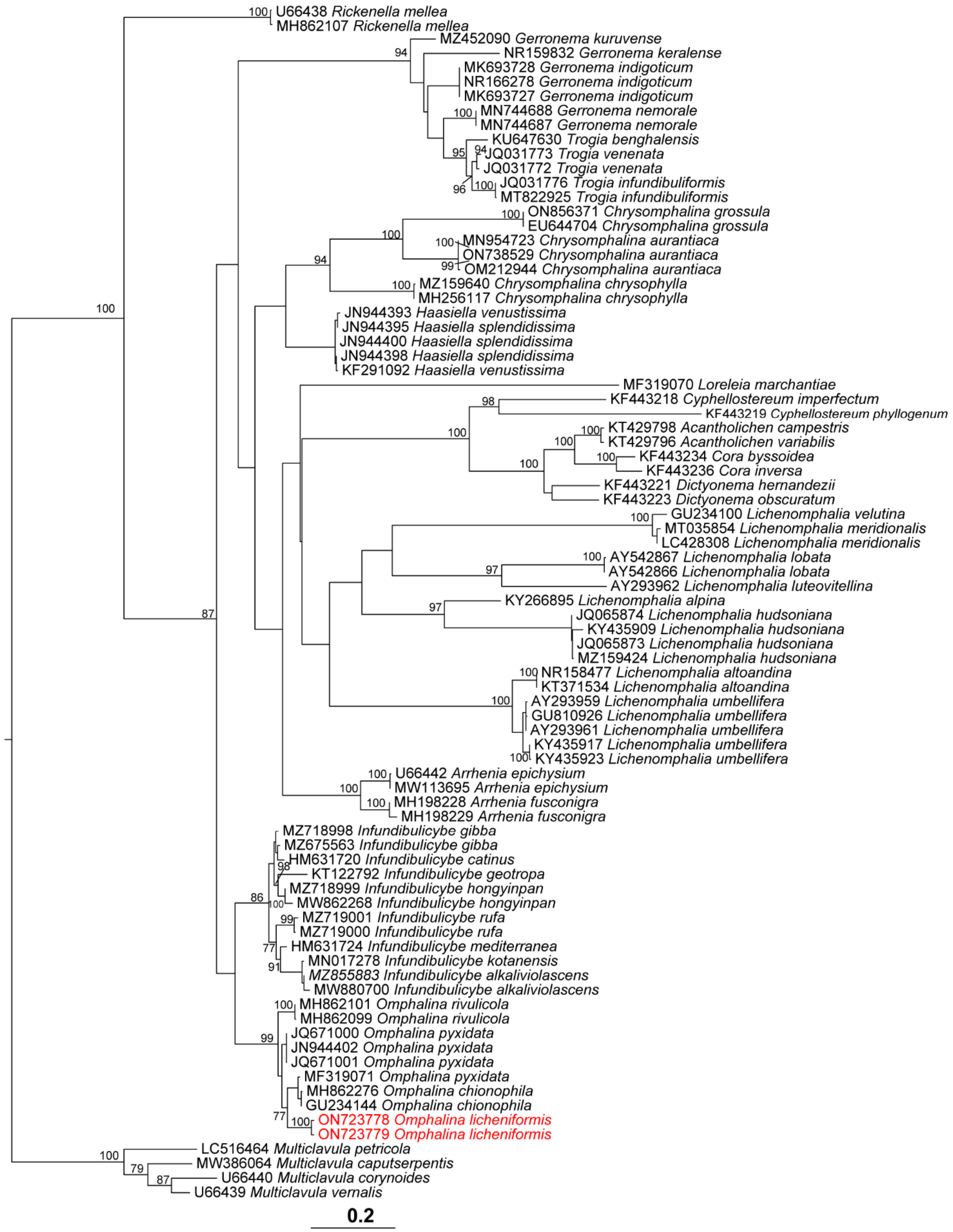


**Figure S2.** The maximum likelihood tree based on the ITS data set. The numbers in each node represent bootstrap support (BS) values. BS values ≥ 75% were plotted on the branches of the tree. The samples in red color indicate that these sequences were newly generated for this study. Scale in 0.2 substitution per site.

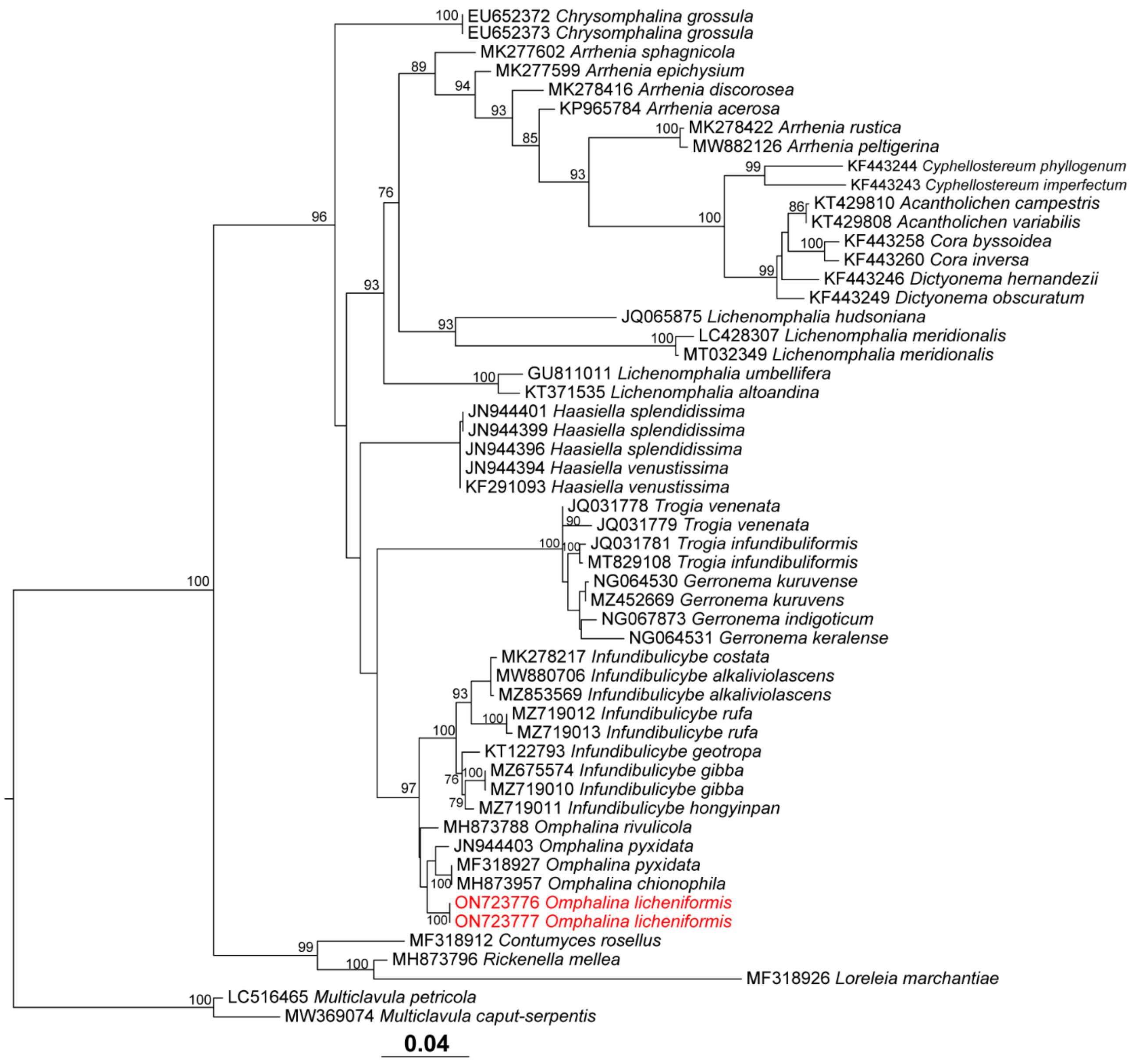


**Figure S3.** The maximum likelihood tree based on the LSU data set. The numbers in each node represent bootstrap support (BS) values. BS values ≥ 75% were plotted on the branches of the tree. The samples in red color indicate that these sequences were newly generated for this study. Scale in 0.04 substitution per site.
